# Deconstructing biomarker generalisation failure in cancer anti-pd1 immunotherapy: toward adaptive patient stratification

**DOI:** 10.1093/bib/bbaf631.076

**Published:** 2025-12-12

**Authors:** Elizabeth Amelia, Chayanit Piyawajanusorn, Pedro Ballester

**Affiliations:** Department of Bioengineering, Imperial College London, UK; HRH Princess Chulabhorn College of Medical Sciences, Thailand; Department of Bioengineering, Imperial College London, UK

## Abstract

**Introduction:**

Predictive biomarkers are central to precision oncology [1], yet transcriptomic signatures for predicting response to Anti-PD1 immunotherapy rarely generalise beyond their discovery cohort. This persistent failure, often attributed to dataset shift, remains poorly understand and hampers clinical translation. We sought to move beyond simple validation and systematically examine the causes of generalisation failure in melanoma and renal cell carcinoma, hypothesising that the brittleness of fitted models is a key barrier.

**Methods:**

We developed 20 gene-panel biomarkers using a consensus [2] pipeline (BioAdapt) across four melanoma and renal cell carcinoma cohorts (N=360). To rigorously test generalisability, each cohort served as both training and independent test data, producing 75 cross-cohort validation experiments. Our framework comprised three stages: (1) diagnosis of dataset dissimilarity using the Maximum Mean Discrepancy test, (2) technical correction via label-free normalisation, and (3) biological stratification of patients by embedding similarity to training cohorts using UMAP.

**Results:**

Direct transfer of models across cohorts yielded near-random performance (mean Matthews Correlation Coefficient ≈ 0). Statistical testing confirmed severe dataset shift (mean Maximum Mean Discrepancy = 0.584). Technical correction modestly improved performance but remained weak. In contrast, biological stratification identified patient subgroups where predictive accuracy nearly doubled compared to corrected models alone, with best-case MCC = 0.41.

**Discussion & Conclusion:**

Our results reveal that biomarker failure is driven less by batch effects than by inherent model brittleness: fitted weights are highly local to the discovery cohort. While technical correction is necessary, stratifying patients by biological similarity enables meaningful recovery of predictive signal. This study establishes a systematic framework for quantifying biomarker limitations and highlights adaptive stratification as a promising route toward subgroup-specific translation in cancer immunotherapy.

**References:**

Piyawajanusorn C., Ghislat G., Ballester P.J. “Predicting atezolizumab response in metastatic urothelial carcinoma patients using machine learning on integrated tumour gene expression and clinical data.” NPJ Precis Oncol. 2025;9(1):170.

Abeel T., Helleputte T., Van de Peer Y., Dupont P., Saeys Y. “Robust biomarker identification for cancer diagnosis with ensemble feature selection methods.” Bioinformatics. 2010;26(3):392–398.

